# Effect of a Six-Week Core Conditioning as a Warm-Up Exercise in Physical Education Classes on Physical Fitness, Movement Capability, and Balance in School-Aged Children

**DOI:** 10.3390/ijerph17155517

**Published:** 2020-07-30

**Authors:** Nai-Jen Chang, I-Hsien Tsai, Chia-Lun Lee, Chun-Hao Liang

**Affiliations:** 1 Department of Sports Medicine, Kaohsiung Medical University, Kaohsiung 807, Taiwan; hsiu920@gmail.com (I.-H.T.T.); u10241059@go.utaipei.edu.tw (C.-H.L.); 2 PhD Program in Biomedical Engineering, Kaohsiung Medical University, Kaohsiung 807, Taiwan; 3 Regenerative Medicine and Cell Therapy Research Center, Kaohsiung Medical University, Kaohsiung 807, Taiwan; 4 Center for Physical and Health Education, National Sun Yat-sen University, Kaohsiung 804, Taiwan; karenlee1129@gmail.com

**Keywords:** pediatrics, trunk muscular endurance, physical activity, sports, functional movement screen

## Abstract

Whether the implementation of feasible, equipment-free, and simple core exercises in warm-up routines in physical education classes for school-aged children is beneficial remains unclear. Therefore, this study investigated the effects of a core conditioning in the warm-up routine of physical education classes on trunk muscular endurance, movement capability, and flexibility in this population. In these pre- and post-test control group experiments, 52 healthy, school-aged children (aged 10–11 years) were cluster randomized allocated to either the dynamic core exercise (DCE) group or general physical education (GPE) group. The DCE group performed a 10-min core exercise routine twice per week for six consecutive weeks; the GPE group performed traditional physical education warm-up exercises regularly. The children were assessed by conducting the trunk muscular endurance test (i.e., dynamic curl-up, static curl-up, plank, and lateral plank), functional movement screen (FMS), and single-leg balance test before and after the intervention. At the end of the intervention, the DCE group demonstrated a significant effect on trunk muscular endurance, movement capability (i.e., FMS scores), flexibility, and balance (each p < 0.001, effect size: 0.38–1.3). Furthermore, the DCE group showed significant improvements in all outcome measurements compared with the GPE group (*p* < 0.05, effect size: 0.29–1.68). These data may provide a reference for incorporating additional core stability exercises in the warm-up routine of physical education classes in school-aged children in the future.

## 1. Introduction

Numerous studies have highlighted that exercises, particularly in physical education classes, in school-aged children have a key effect on their development [1,2,3]. Before initiating main courses in physical education classes, warm-up routines should be implemented. Traditionally, several minutes of light-intensity of active range-of-motion exercises involving the upper and lower extremities followed by static stretching are recommended for young participants. Although static stretching increases flexibility, it may also impair athletic performance [4,5,6]. Dynamic warm-up exercises include running, hops, jumps, skips, and various movement-based exercises for the upper and lower body, and they are recommended before initiating training [7]. The benefits of dynamic warm-up exercises include enhancing a range of motions, increasing core body temperature, elevating motor unit excitability, and improving movement capability [7,8,9,10]. Faigenbaum et al. demonstrated that 10-min dynamic warm-up exercises significantly increase vertical jump and shuttle-run performances in children (mean age of 11 years) [11].

In the human body, core muscles include global axial stabilizers, such as rectus abdominis, external oblique abdominis, erector spinae, gluteus, and local axial stabilizers, such as multifidus, transverse abdominis, and internal oblique abdominis [12]. These trunk muscles play an important role in controlling movements and maintaining core stability [13]. Core stability encompasses all facets of muscle strength, endurance, and conditioning that are required for controlling the movements in the lumbo-pelvic-hip complex [14]. Therefore, to enhance core stability effectively, the exercise program should consider global muscle and synergistic muscle groups that are relevant to the kinetic chains rather than trunk extensors alone, especially in school-aged children [15]. Additionally, good core stability results in better athletic performance [16] and better posture control and balance [17]. However, in practical applications, isolating the trunk muscles (e.g., back extensors) is a challenge because of the requirement for special equipment for pelvic stabilization [18] or the use of a balance board and stability disc exercises [19]. Core training in youth is an important facet of maintaining musculoskeletal health, improving bone health, and reducing the risk of sports-related injuries [15,20,21]. Higher core stability may enhance exercise performance by ensuring greater force production in upper and lower extremities [19]. Therefore, the core stability program increased muscular performance, promising for possible implementation into physical education classes in school-aged children.

Performing high-quality functional movements is associated with better athletic performance and lower risk of sports injuries [22,23]. Functional movement screen (FMS) is used for the objective screening of core muscle strength, flexibility, coordination, and balance [24,25]. FMS includes a series of seven fundamental movement patterns, namely deep squat (DS), hurdle step (HS), in-line lunge (ILL), shoulder mobility (SM), active straight leg raise (ASLR), trunk stability push-up (TSPU), and rotary stability (RS). Each of the seven screens was scored on a 0–3 scale based on specific objective criteria [26]. This tool offers high intertester and intratester reliability in specific functional movement patterns [27,28]. Mitchell et al. demonstrated that core strength was positively correlated with the total FMS score (r = 0.31; *p* = 0.006) in children aged 8–11 years [29], thus suggesting that FMS is a feasible option in assessing the fitness in children.

Implementing highly feasible, equipment-free, and simple core routines in physical education warm-up classes is difficult and critical in increasing trunk muscular endurance and improving movement capability and flexibility in school-age children. In field practice, unlike adults, school-aged children are less likely to perform additional core-training exercises in a gym or by using specialized equipment. Additionally, children’s trunk muscles are prone to be underdeveloped due to the lack of additional training during typical physical education classes or activities [15]. However, no study has investigated this issue. Therefore, this study investigated the effects of a core conditioning in the warm-up routine of physical education classes on trunk muscular endurance, movement capability, and flexibility in school-aged children. We hypothesized that the core conditioning program would significantly improve the outcomes in most of the assessment items adopted in the study.

## 2. Materials and Methods

### 2.1. Participants

Fifty-two fourth-grade children were recruited from a national elementary school located in Kaohsiung City in Taiwan. The physical education teacher conducted pre-designed courses for these 52 children (28 girls and 24 boys, aged 10–11 years). An independent researcher who was not involved in the recruitment prepared cluster random allocation cards based on classes (A class: Dynamic core exercise (DCE) group; B class: General physical education (GPE) group) and placed them in sealed, opaque envelopes. A researcher drew and opened the envelope and notified the group of assignment. However, blinding the children to the group assignment was impossible. Children were allocated to either the DCE group or GPE group. Exclusion criteria included the presence of cardiovascular or respiratory diseases; contraindications to exercise (e.g., neuromusculoskeletal injury); muscle strains and ligament sprains; joint instability or laxity in lower extremities; head or spinal injury; visual, vestibular, or balance disorders in the preceding six months; and refusal of a child or corresponding parents to participate. The study protocol was approved by the institutional review board (KMUHIRB-SV(I)-20180083 and approval dated: 2019/03/08). All the participants were informed regarding the benefits and risks of the investigation. Written informed consent was obtained from the children and their parents or guardians before data collection. At the six-week follow-up, all children completed this trial.

### 2.2. Study Procedures

This study was a pre- and post-test control group experimental design to determine if a 10-min core stability program could increase trunk muscular endurance, movement capability, and flexibility. Both the groups performed interventions at their school. Prior to the assessment, the children attended a session to familiarize themselves with the exercises, in which they were instructed by the same physical education teacher on how to perform the core exercise program and informed about the assessment protocols. The physical education teacher trained the children on how to correctly accomplish each movement and provided verbal feedback as required during assigned practice time. At the time of explaining the experiment to the participants, we also familiarized them with the procedures and assessment tools (e.g., trunk muscular endurance, FMS, and flexibility tests) of the study for practice. The students practiced each testing and exercise item 10 min before starting physical education classes over one week (approximately two classes). After the familiarization session, before initiating the six-week intervention, baseline data were collected from each participant. Before each test session, the children were requested to avoid strenuous activities for 24 h. After completing the pre-test assessments, the children performed either DCE or GPE activities for six weeks. The exercise protocols were provided in detail later. At the end of testing, post-test assessments were performed in the same order as the pre-test measures in the next class after the intervention. Trained and blinded graduate students from the department of sports medicine science executed all tests to ensure testing consistency and reduce bias.

### 2.3. Exercise Protocols

Physical education classes were conducted twice a week for six weeks by the same physical education teacher. The course comprised 10-min warm-up, 25-min formal physical education, and 5 min cool-down over 40 min per class. The formal physical education and cool-down programs were the same in both groups.

#### 2.3.1. Dynamic Core Exercise (DCE) Group

In addition to their regularly scheduled physical education content, the children in this group performed dynamic core stability exercises for six weeks. The exercises were selected and modified based on prior works [15,21,30]. A total of 12 movements encompassing whole body movements were performed as part of the warm-up routine and included pseudo-jump rope, high knees, lunges, burpees, Heisman exercise, low plank, mummy kick, walking hands, vertical jump-and-hold, planks, single-legged jump-with-hold, and calf raises (Table 1). Each movement was performed for 30 s with a 10-s rest interval, twice per week for six consecutive weeks. The physical education teacher provided individualized feedback to ensure good quality of postural alignment. If a student could not execute the plank (i.e., hold the body weight), the teacher provided a modified plank exercise that included forearm and knee support instead of forearm and forefoot support alone. If one-leg calf raise could not be performed, then both legs were adjusted to avoid knee valgus or compensatory bending of the trunk to the side. The movement could be slightly adjusted according to student’s ability to ensure good quality of exercise and avoid injuries.

#### 2.3.2. General Physical Education (GPE) Group

The GPE group was the control group. The warm-up routine included 3 min jogging, a general active range of motion exercises, and 10 min static stretching for upper and lower extremities, twice per week consecutively for six weeks.

### 2.4. Measures

#### 2.4.1. Trunk Muscular Endurance Assessments

Four tests were selected because they activate trunk and core muscles. The plank test required the children to maintain 90° between the trunk and elbows. Only the elbows and toes were allowed to touch the mat. Additionally, adjustable movements, if any, must be corrected within 3 s; otherwise, the assessment was terminated. The total time recorded while maintaining the proper position was included in the analysis.

In the lateral plank test, the children were required to maintain a linear form from the head to foot while taking support of the right elbow placed on the mat. Only the right elbow and right shoe were allowed to be in contact with the mat. Additionally, corrections had to be made within 3 s; otherwise, the assessment was terminated. The total time recorded while maintaining the proper position was included in the analysis.

The dynamic curl-up test was based on FITNESSGRAM’s guidelines (https://fitnessgram.net/). The children were required to lay on the mat with their knees bent, hands placed on both sides of the body, and actively curl up and down while sliding their fingers across a distance of a 4.5-inch-wide strap at a specific tempo. On each curl-up, their backs and heads must touch the mat. The assessment was terminated if two errors were noted. The total number of repetitions was included in the analysis.

The static curl-up test was performed according to the methods reported in a previous study [15]. The same principles as those for the dynamic curl-up test were incorporated. The children were required to maintain their trunk at a 45° upward position, with both hands straight so that the fingers are as far as possible at the end of the 4.5-inch-wide tape (the ends where the fingers touched after rolling up the trunk). Once a finger is moved away from the end of the tape, it must return to the correct position within 3 s; otherwise, the assessment was terminated. The total duration for which the proper position was maintained was included in the analysis.

#### 2.4.2. Functional Movement Screen (FMS) Testing

**Figure 1:**
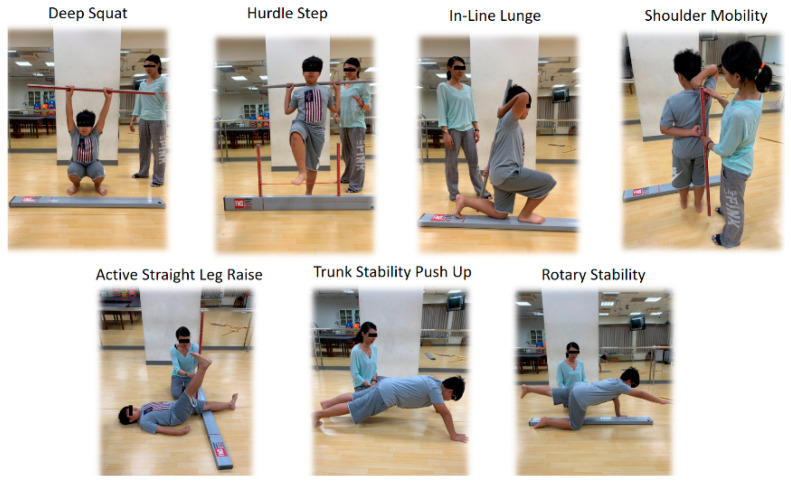
Function movement screen testing.

FMS included seven fundamental movement tasks and three clearance tests (Figure 1). It had high intertester (0.843) and intratester reliability (0.869) based on the intraclass correlation coefficients [31]. Each FMS was scored using an ordinal scale (0–3) to obtain a composite score (0–21); 0 indicated any occurrence of pain during any movements, and 3 indicated that the movement was performed correctly without any compensations. Higher total scores indicated better movement capability [32]. Regarding the seven movement tasks, the children were blinded to the assessors’ scores by the children’s DS, HS, ILL, SM, ASLR, TSPU, and RS performances. Only simple verbal instructions without any coaching were allowed during the FMS testing process. Regarding the three clearance tests, the children were assessed for any pain during the movements of the shoulder impingement, spinal flexion, and spinal extension tests.

#### 2.4.3. Sit-And-Reach Test

The standard sit-and-reach test was conducted to assess the flexibility of children’s hamstrings and lower backs [33,34]. The children were instructed to sit with their knees fully extended with the soles flat against the test device and shoulder width apart. Then, they were instructed to place one hand over another and slowly reach up along the measurement scale as far as possible; subsequently, they were instructed to hold the position for 2 s, while maintaining their knees without any bending. Higher scores indicated better performance. Two trials were performed. The most distant point the fingertips could reach was recorded for the analysis.

#### 2.4.4. Balance Test

**Figure 2:**
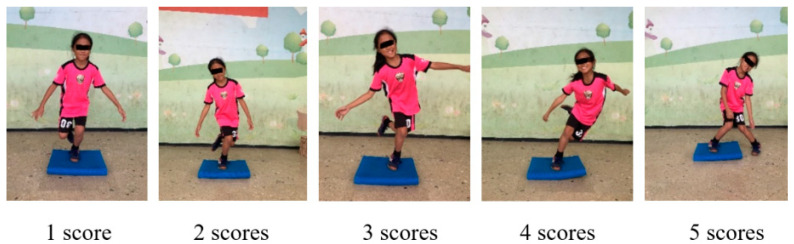
Scoring scale for single-leg balance test.

The single-leg balance test for children was based on a previously reported test [35]. This test was also a neuromotor screening test. To reduce assessment bias, a blinded investigator measured all the movements of the children by using video recordings. The camera was set before the children. Each child was asked to execute a single-legged stance on the balance pad. Once a child could maintain their balance, the child was asked to squat to a knee flexion angle of 90° and rise back to full extension while maintaining the trunk upright throughout the process. The ability to maintain balance in addition to the knee valgus or knee varus alignment were assessed and recorded. These observations were scored from 1 (normal) to 5 (worst). A score of 1 indicates perfect balance, 2 indicates poor hip control with dropped hip and slight valgus of the tested knee, 3 indicates using upper extremities to maintain balance and upper trunk motion, 4 indicates substantial upper extremity and trunk movements outside the central balance line and an approximate failure, and 5 indicates failure with the other foot touching the ground (Figure 2). Each child performed the test thrice, and the best outcome was recorded and given single-blinded analysis by the same assessor.

### 2.5. Statistical Analyses

All data analyses were performed using SPSS, version 20 (IBM Inc., Armonk, NY, USA). Data are presented as median [interquartile range (IQR)]. Data were assessed visually and statistically for normality (Shapiro–Wilk test, *p* > 0.05); however, if the variables were not normally distributed, a nonparametric Mann–Whitney U test was performed for intergroup comparisons. A nonparametric Wilcoxon-signed rank test was performed for intragroup comparisons. The significance level (α) was considered *p* < 0.05. Additionally, to present the magnitude of the effect, the effect size (ES, Cohen’s d), which is the difference between pre-test and post-test means divided by their common SD, was calculated and interpreted as small (d = 0.2), medium (d = 0.5), or large (d = 0.8). With the alpha level set at 0.05, post hoc power analysis was conducted to calculate the sample size and effect size with G*Power software [36].

## 3. Results

All children in both the groups completed the program, and their data were analyzed (Table 2). The results of all outcomes are summarized in Table 3 and Table 4. No significant differences were observed between the DCE and GPE groups in the baseline data, including the dynamic curl-up, static curl-up, plank, side plank, FMS total scores, flexibility, and balance tests. No physical injury was caused by the core conditioning exercises in this study.

The DCE group demonstrated significant improvements in all outcome measurements (*p* < 0.001) compared with the baseline data. The effect sizes (Cohen’s d) were 0.79 for the dynamic curl-up test, 0.97 for the static curl-up test, 0.91 for the plank test, 1.15 for the side plank test, 1.3 for the FMS total scores, 0.38 for flexibility test, and 1.05 for balance test. The results demonstrated that the DCE group had a greater effect on all muscular fitness test items, FMS total score, and balance tests. The GPE group had significant improvements in the side plank test (*p* = 0.001), FMS total scores (p < 0.001), and flexibility test (*p* = 0.012). Conversely, the GPE group had significantly decreased values in the static curl-up (*p* < 0.009) and plank (*p* = 0.001) test when compared with the baseline data. Furthermore, the post hoc power analysis was performed using effect size between time points on each parameter (Table 3). Here, power analysis resulted in high power values from 99% to 100% in the DCE group and from 24% to 99% in the GPE group (Table 3), indicating a sufficient sample size to detect the change difference. Additionally, the DCE group significantly improved regarding all outcome measurements compared with the GPE group (*p* < 0.05, effect size: 0.29–1.68) (Table 4).

## 4. Discussion

To the best of our knowledge, this is the first study to investigate the effects of performing dynamic core routines in physical education classes on trunk muscular endurance, movement capability, flexibility, and balance in school-aged children. Our results indicated that a six-week DCE program resulted in significantly superior improvements on all muscular fitness test items, FMS total scores, flexibility, and balance tests. Furthermore, the overall interventional effectiveness was significantly higher in the DCE group than in the GPE group, thus suggesting that the DCE intervention has a greater effect.

The core stability affects the effective use of the strength, power, and endurance required. A weak core is a fundamental problem of inefficient movements that ultimately results in injuries [16].

In contrast to previous studies on core strength training programs conducted among pediatric age groups, we performed this experimental control study on school-aged children and required minimal time and no equipment to enhance outcomes efficiently. Ozmen et al. found that a six-week core strength training program significantly improved balance and core endurance [37]. However, a limitation of their study was that only adolescent badminton players were recruited. Additionally, in that study, physioball was vital to the interventional training program, which may be relatively difficult to incorporate into physical education classes. Oliver et al. implemented a core stability program in elementary physical education curriculum for a period of 10 months and found that the intervention improved core strength and endurance [21]. A limitation of their study, however, was that a long training period was required to produce the effects. Additionally, Allen et al. integrated a six-week core stability program into the physical education classes of school children (average age of 11 years), which was effective in improving trunk and core muscular endurance [15]. A limitation of their study, however, was the absence of a control group to confirm the true benefits of such an intervention program. Additionally, how these core exercise programs affect other parameters, such as movement capability, flexibility, and balance, is unclear.

The dynamic core stability intervention can be beneficial from the implementation of dynamic whole-body movements rather than trunk muscles alone. Additionally, such a moderate intensity warm-up can engage the major muscles for more vigorous movements by increasing the core body temperature and motor unit recruitment [20]. In the present study, the DCE group exhibited considerable improvements in all trunk muscular endurance assessments (all *p* < 0.001, effect size: 0.38–1.3), indicating enhancement of core stability. However, we cannot rule out the possibility that the measurements were influenced by learning effects associated with the involved movements (i.e., plank exercises). Furthermore, our findings demonstrated that the FMS scores and single-leg balance test results were considerably better following DCE (*p* < 0.001, ES = 1.3; *p* < 0.001, ES = 1.05; respectively). Furthermore, the DCE group showed significant improvements with moderate-to-large effects in movement capability and balance tests compared with the control group (*p* = 0.004, ES = 0.51; *p* = 0.04, ES = 0.86; respectively). This indicates that children can increase their core strength and endurance over time, enabling them to maintain postural control, reduce inefficiency in their movement patterns [29], and improve balance stabilization [38]. We postulated that the positive effects of DCE on movement capability (i.e., FMS scores) and physical performance (i.e., single-leg balance) can be explained by the specific role of the trunk as a kinetic link that facilitates the transfer of torque and angular movement between upper and lower extremities during whole body movements as part of fitness activities and activities of daily living [39,40].

Interestingly, the GPE group displayed improvements in the sit-and-reach test, lateral plank test, and FMS total scores. In contrast to previous studies, our study included a control group (i.e., GPE group) to verify the effects of the intervention. Our data indicated that including only a GPE warm-up routine may be suboptimal for improving physical fitness in school-aged children. Furthermore, in this study, the GPE group performed routine jogging, light intensity of general active range-of-motion exercises, and static stretching for upper and lower extremities. We postulated that these may result in unanticipated adaptations to one’s flexibility and core stability. Therefore, this is not to say that the general warm-up protocol should be completely replaced by dynamic core stability exercises in a child’s pre-event program but rather that physical education teachers should consider the potential additional effects on exercise performance.

For practical application, we recommend that school-aged children begin their exercise program with a 10-min warm-up DCE routine (twice a week for six weeks), which requires minimal time and equipment to complete. This program is designed to train the major trunk and lower extremity muscles necessary for motor competence and functional performance. This program can potentially be adapted to children of other age groups and may considerably improve children’s physical fitness, which is particularly imperative given the global crisis of pediatric obesity.

## 5. Conclusions

A six-week DCE program as a warm-up exercise significantly improved trunk muscular endurance, movement capability, flexibility, and balance in school-aged children. These results may provide a reference for the incorporation of core stability exercises as part of a warm-up routine for school-aged children in physical education classes. The exercise can feasibly be performed in physical education classes. Future studies should investigate the long-term effects of this program on a larger population.

## Figures and Tables

**Table 1:** Dynamic core stability program.

Movements	Exercise Description and Instructions
Pseudo-jump rope	Stand upright, abdomen tucked in, rotate arms to mimic jump rope, jump in the same plane alternating the legs with each jump incorrect movements to be avoided: jumping with one leg forward and one leg behind, trunk leaning forward
High knees	Stand upright with one arm lifted 90° from the body; raise one knee high and bring it back down again. Repeat with the opposite knee and alternate the arm raised incorrect movements to be avoided: uneven high knees, foot touching the hand while arm is at an angle less than 90° from the body, trunk leaning forward
Lunges	Stand upright with hands on the hips; take a step and bend that knee to 90° while keeping the body upright. Keep feet aligned with the sagittal plane. incorrect movements to be avoided: the front knee in valgus, trunk leaning forward or toward one side
Burpees	Stand upright, squat, and place hands on the floor. Kick feet back into the high plank position, return to the squat position, and then jump incorrect movements to be avoided: trunk not in the plank position
Heisman exercise	Take three small steps alternating high knees while balancing and keeping the abdomen tight incorrect movements to be avoided: loss of balance, relaxation of the abdomen, trunk leaning forward
Low plank	Facing downwards, lean on the elbows with feet shoulder-width apart and only toes touching the ground; maintain a straight line down the shoulder, trunk, and buttocks incorrect movements to be avoided: shoulder or buttocks elevated above the trunk
Mummy kick	Standing upright with arms straight in front of the body and parallel to the ground at shoulder height, alternate left and right leg kicks and cross arms while keeping abdomen tight incorrect movements to be avoided: bend elbow, lock knees
Walking hands	Starting in a high plank position, move hands and legs to the low plank position and back to the high plank position 3 times incorrect movements to be avoided: loss of abdominal control while moving the hands and legs to the low plank position
Vertical jump-and-hold	With trunk slightly bent, one hip and knee in flexion, and feet shoulder-width apart, jump vertically. Hold this position until landing without losing balance incorrect movements to be avoided: trunk bent, knee locked, knee in valgus or varus while landing
Planks	Facing downward with palms on the floor and feet shoulder-width apart with only toes touching the ground, maintain a straight line across the shoulder, trunk, and buttocks incorrect movements to be avoided: shoulder or buttocks elevated above the trunk
Single-legged jump-with-hold	In an upright position standing on only one leg with the raised knee in slight flexion, jump and hold this position until landing without losing balance incorrect movements to be avoided: trunk bent, knee locked, knee in valgus or varus
Calf raises	In an upright position with hands touching the wall, raise heels up to the maximal range of motion and then slowly back toward the floor incorrect movements to be avoided: bending the knee while raising the heel, lowering the heel without control

**Table 2:** Baseline characteristics of the dynamic core exercise and general physical education groups.

Characteristic	Dynamic Core Exercise (N = 27)	General Physical Education (N = 25)
Boy/girl (n)	13/14	11/14
Age (year)	11 (10,11)	10 (10,11)
Height (cm)	141.4 (136.25,145)	138.2 (135.9,143.8)
Body weight (kg)	40.5 (30.35,46.45)	32.5 (28.1,41.9)
Body mass index (kg/m^2^)	19.8 (15.85,23.70)	16.4 (14.9,19.8)

Unless specified, data are presented as median (interquartile range (IQR)).

**Table 3:** Pre-test and post-test descriptive results.

Measurements	Pretest	Posttest	Effect Size	Intra-Group *p*	Power
**Dynamic core exercise group (N = 27)**					
Dynamic Curl Up (reps)	14 (10,21)	23 (14.5,27)	0.79	<0.001	0.98
Static Curl Up (sec)	75 (46,117.5)	138 (100,240)	0.97	<0.001	0.99
Plank (sec)	83 (68,115)	130 (100.5,178.5)	0.91	<0.001	0.99
Lateral Plank (sec)	23 (13.5,46)	55 (37.5,70)	1.15	<0.001	0.99
FMS total scores	14 (11.5,15)	16 (15,17)	1.3	<0.001	1
Flexibility (cm)	26 (22,33.5)	29 (25.5,36.5)	0.38	<0.001	0.99
Balance (scores)	3 (3,5)	2 (2,3)	1.05	<0.001	0.99
**General Physical Education Group (N = 25)**					
Dynamic Curl Up (reps)	12 (8,21)	17 (12,22)	0.38	0.069	0.60
Static Curl Up (sec)	95 (80,145)	78 (48,115)	0.44	0.009	0.56
Plank (sec)	80 (65,113)	59 (73,74)	0.8	0.001	0.99
Lateral Plank (sec)	22 (14,40)	46 (28,61)	0.88	<0.001	0.99
FMS total scores	13 (12,14)	15 (13,17)	0.79	<0.001	0.99
Flexibility (cm)	27 (24,31)	25 (22,30)	0.23	0.012	0.86
Balance (scores)	3 (2,5)	3 (2,4)	0.18	0.314	0.24

Data are presented as median (interquartile range (IQR)). Wilcoxon-signed rank test was performed for intragroup comparisons. P, significant difference (*p* < 0.05) compared with pre-test data. Effect sizes (d = M1—M2/σpooled) were considered small if d = 0.2, moderate if d = 0.5, and large if d = 0.8.

**Table 4:** Changes in trunk muscular endurance, movement capability, flexibility, and balance after six-week dynamic core exercise (DCE) and general physical education (GPE).

Measurements	Changes in DCE Group	Changes in GPE Group	Effect Size	Between-Group *p*
Dynamic Curl Up (reps)	6 (2,12)	4 (−2,8)	0.58	0.043
Static Curl Up (sec)	76 (40,113.5)	−33 (−71,5)	1.18	<0.001
Plank (sec)	46 (28.5,83)	−26 (−43,−6)	1.68	0.001
Lateral Plank (sec)	31 (12,39)	22 (6,28)	0.31	<0.001
FMS total scores	2 (1,3)	1 (0,3)	0.51	0.044
Flexibility (cm)	2 (1,4.5)	−2 (−4,1)	0.29	<0.001
Balance (scores)	−1 (−2,−0.5)	0 (−1,0)	0.86	0.04

Data are presented as median (interquartile range (IQR)). Mann–Whitney U test was performed for intergroup comparisons. *p*, significant change difference between groups. Effect sizes (d = M1—M2/σpooled) were considered small if 0.2, moderate if 0.5, and large if 0.8.
